# Optimizing Prehospital Stroke Systems of Care-Reacting to Changing Paradigms (OPUS-REACH): a pragmatic registry of large vessel occlusion stroke patients to create evidence-based stroke systems of care and eliminate disparities in access to stroke care

**DOI:** 10.1186/s12883-022-02653-x

**Published:** 2022-04-07

**Authors:** Derek L. Isenberg, Kevin A. Henry, Adam Sigal, Traci Deaner, Jason T. Nomura, Kathleen A. Murphy, Derek Cooney, Susan Wojcik, Ethan S. Brandler, Alexander Kuc, Gerard Carroll, Chadd Krauss, Judy B. Shahan, Joseph Herres, Daniel Ackerman, Nina T. Gentile

**Affiliations:** 1 Department of Emergency Medicine, Lewis Katz School of Medicine at Temple University, Philadelphia, PA USA; 2 Department Geography and Urban Studies, Temple University, Philadelphia, PA USA; 3 Department of Emergency Medicine, Tower Health, Reading, PA USA; 4 Department of Emergency Medicine, ChristianaCare, Newark, DE USA; 5 Department of Emergency Medicine, State University of New York-Upstate Campus, Syracuse, NY USA; 6 Deparement of Emergency Medicine, Stony Brook Medicine, Stony Brook, NY USA; 7 Department of Emergency Medicine, Cooper Medical School of Rowan University, Camden, NJ USA; 8 Department of Emergency Medicine, Geisinger, Danville, PA USA; 9 Department of Emergency Medicine, Einstein Healthcare Network, Philadelphia, PA USA; 10 Department of Neurology, St. Luke’s University Health Network, Bethlehem, PA USA

**Keywords:** Ischemic stroke, Large vessel occlusion mechanical thrombectomy, Endovascular, Emergency medical services, Prehospital, Systems of care

## Abstract

**Background:**

Large vessel occlusion (LVO) strokes are best treated with rapid endovascular therapy (EVT). There are two routes that LVO stroke patients can take to EVT therapy when transported by EMS: primary transport (ambulance transports directly to an endovascular stroke center (ESC) or secondary transport (EMS transports to a non-ESC then transfers for EVT). There is no clear evidence which path to care results in better functional outcomes for LVO stroke patients. To find this answer, an analysis of a large, real-world population of LVO stroke patients must be performed.

**Methods:**

A pragmatic registry of LVO stroke patients from nine health systems across the United States. The nine health systems span urban and rural populations as well as the spectrum of socioeconomic statuses. We will use univariate and multivariate analysis to explore the relationships between type of EMS transport, socioeconomic factors, and LVO stroke outcomes. We will use geographic information systems and spatial analysis to examine the complex movements of patients in time and space. To detect an 8% difference between groups, with a 3:1 patient ratio of primary to secondary transports, 95% confidence and 80% power, we will need approximately 1600 patients.

The primary outcome is the patients with modified Rankin Scale (mRS) ≤ 2 at 90 days. Subgroup analyses include patients who receive intravenous thrombolysis and duration of stroke systems. Secondary analyses include socioeconomic factors associated with poor outcomes after LVO stroke.

**Discussion:**

Using the data obtained from the OPUS-REACH registry, we will develop evidence based algorithms for prehospital transport of LVO stroke patients. Unlike prior research, the OPUS-REACH registry contains patient-level data spanning from EMS dispatch to ninety day functional outcomes. We expect that we will find modifiable factors and socioeconomic disparities associated with poor outcomes in LVO stroke. OPUS-REACH with its breadth of locations, detailed patient records, and multidisciplinary researchers will design the optimal prehospital stroke system of care for LVO stroke patients.

**Supplementary Information:**

The online version contains supplementary material available at 10.1186/s12883-022-02653-x.

## Background

In the United States, approximately 800,000 people experience a stroke each year [1]. Stroke causes one in six deaths from cardiovascular disease and is a leading cause of disability in the United States [2, 3]. Large vessel occlusion (LVO) strokes account for 11-31% of acute ischemic strokes (AIS) [4]. For LVO strokes, endovascular treatment (EVT) results in better outcomes than intravenous thrombolysis (IVT) alone [4–9].

A recent study found that LVO strokes patients were over 3.5 times more likely to undergo EVT if they presented at an endovascular capable stroke center (ESC) compared to a non-ESC [10]. In addition, for every 15 min delay in EVT up to 270 min, there is a 1% worsening of good functional outcomes [11]. Hospital costs and lengths of stay are increased when patients are transferred for EVT compared to patients directly transported to an ESC [12]. Patients who require transfer for EVT are less likely to be discharged home and ambulate independently than patients transported directly to an ESC [13].

**Fig. 1:**
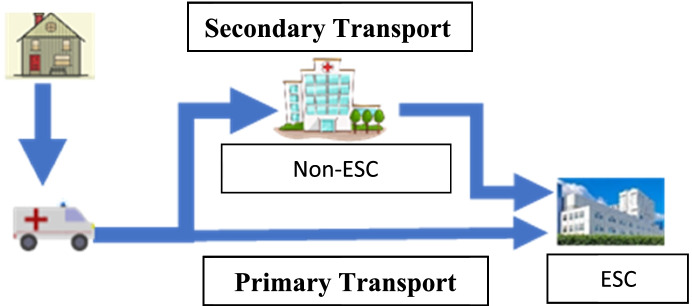
Primary versus secondary stroke transport

There are two routes that LVO stroke patients can take to EVT therapy when transported by EMS: 1) transport a suspected LVO stroke patient directly to an ESC (primary transport) or 2) transport a patient to a non-ESC then transfers the patient to an ESC for EVT (secondary transport) (Fig. 1). EMS could transport every suspected LVO stroke patients to an ESC. However, there are costs to the healthcare system if an ambulance bypasses a non-ESC for an ESC. In a rural area, a longer transport duration may take an ambulance out of service for a significant amount of time. If a strategy of primary transport is undertaken for all suspected LVO stroke patients, patients will be over-triaged to ESCs when they do not require advanced resources. One study estimated an approximately 6-19% over-triage rate to CSC for LVO stroke [14].

There is scant evidence examining the relationship of primary and secondary transport to ESC to functional outcomes. Several authors have attempted to address the design of stroke systems of care through modeling. Two studies have suggested that suggested that EMS should bypass community hospitals if the ESC is within a 60-min transport time, while another study suggested that EMS bypass community hospitals if within 20 min of an ESC [15–17]. One patient level study found an 8% increase in good functional outcomes when patients were transported directly to an ESC compared to those patents transferred for EVT [15]. However, this study was conducted in the context of a clinical trial where stroke processes were optimized. In addition, bypass times were estimated based on driving distances and a fictional on-scene time.

To develop an evidence based algorithm for the transport of LVO stroke patients, an analysis of factors associated with functional outcomes needs to be performed on a large, real world population of LVO stroke patients. The Optimizing Prehospital Stroke Systems of Care-Reacting to Changing Paradigms (OPUS-REACH) registry is designed to answer this question.

## Methods and design

### Design

The Optimizing Prehospital Stroke Systems of Care-Reacting to Changing Paradigms (OPUS-REACH) registry will include information from nine health systems across the Northeastern United States. The registry includes data from EMS dispatch to 90 day outcome.

### Participating sites

**Fig. 2:**
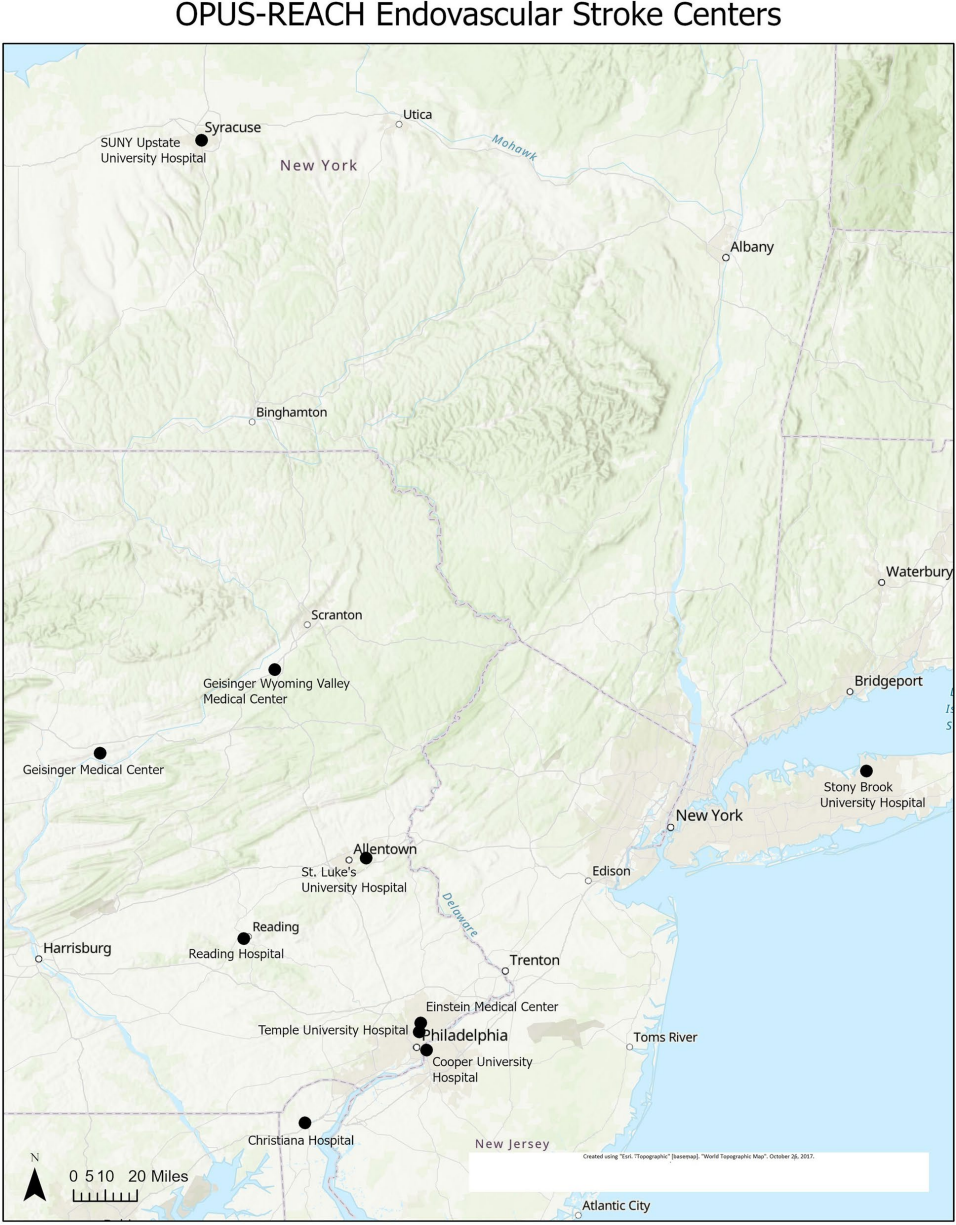
Map of endovascular stroke centers participating in OPUS-REACH

**Fig. 3:**
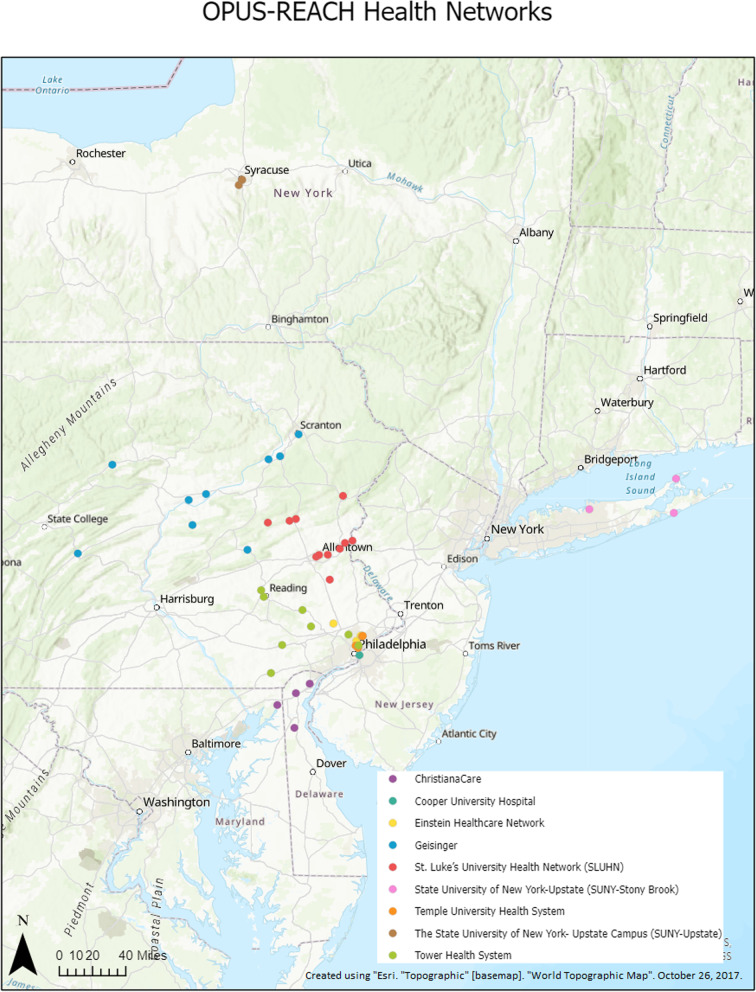
Map of all acute care hospitals participating in OPUS-REACH

The OPUS-REACH consortium consists of nine health systems with the shared goal of studying and optimizing prehospital systems of care for LVO stroke patients (Table 1). These nine health systems consist of 46 acute care hospitals including 10 ESCs and span systems span urban, suburban, and rural areas (Figs. 2 and 3). The nine health systems account for over 1.7 million ED visits, over 10,000 stroke admissions, and approximately 850 mechanical thrombectomies annually (Supplement 1). The lead hospital in the OPUS-REACH network, Temple University, is an innovator in using Geographical Information Systems (GIS) to assess systems of healthcare delivery.

**Table 1:** Summary of health system data from OPUS-REACH

Health System	Acute Care Hospitals	Emergency Department Volume	Stroke Admissions	EVT procedures for acute stroke
**Temple University Health System**	3	200,000	500	40
**Einstein Healthcare Network**	3	162,000	620	40
**Cooper University Hospital**	1	80,000	600	140
**Tower Health System**	7	221,755	725	55
**Geisinger**	12	300,000	2000	160
**State University of New York-Stony Brook**	3	124,610	1000	200
**St. Luke’s University Health Network**	12	390,000	1800	83
**ChristianaCare**	3	200,000	1500	150
**State University of New York- Upstate Campus**	2	111,000	1900	90
**Totals**	46	1,789,445	10,645	858

### Patient population and eligibility

Our LVO stroke registry will include all patients from the nine health systems who have undergone EVT for LVO stroke between 2015 and 2020. Patients will be excluded if they were not transported to either the ESC or non-ESC by EMS or the patient does not have a 90 day functional outcome assessed.

### Study procedures

Each spoke in the OPUS-REACH consortium has a site investigator (SI) responsible for oversight of data collection. These SI are primarily emergency physicians, but also include neurologists. Data collection for the OPUS-REACH registry will be extracted at each site individually and then submitted to a central registry housed at Temple University [18]. The REDCAP database is approved for the storage of protected health information. Only study personnel approved by the Institutional Review Board and designated by the principal investigator (DI) will have access to protected health information. All investigators listed on this manuscript will have access to de-identified data for the purpose of writing manuscripts for publication. Authorship will be determined based on the contributions of each investigator to the manuscript. Data and results will be disseminated via peer-reviewed journals in the fields of neurology, emergency medicine, and public health.

### Outcomes

The main outcome variable is functional status at 90 days as measured by the Modified Rankin Scale (mRS) [19–22]. Scores of 0 to 2 are considered good outcomes while scores of 3 to 6 are considered poor outcomes. In the cases where a 90-day mRS may not be available, the SI from each site will review the electronic medical record for clinic notes, emergency department visits, or physical therapy notes, that would describe the patient’s functional outcome at 90 days. They will then assign a score of “good” or “poor functional outcome.” This approach has been documented as an accurate way to estimate a patient’s functional outcome [22].

We will compare outcomes between patients transferred for EVT to those directly transported by EMS to an EVT center. We will stratify patients by eligibility for IVT as well as onset of stroke. We will also stratify patients by drive times, based on modeling, to the EVT center.

### Data capture

Hospitals will identify LVO stroke patients treated with EVT at their facilities. For each patient who underwent an EVT, the SI will complete a standardized case report form (CRF). This CRF will include prehospital data, in-hospital data, and 90 day functional outcomes.

### Sample size

A prior study showed an 8% absolute difference in outcomes for LVO stroke patients between patients who were transported directly to an ESC versus those who were transferred from a primary stroke center: 40% in the direct transport group v. 32% in the transfer group [21]. Therefore, to detect an 8% difference between groups, with a 3:1 patient ratio of primary to secondary transports, 95% confidence and 80% power, we will need approximately 1600 patients.

### Statistical and spatial analysis

The use of GIS and spatial analysis moves prehospital system design from expert opinion to a data-driven process. Geocoding patient data (e.g., pick up locations), integrating it into a GIS, and linking it with other clinical data will provide the necessary platform to examine the complex movements of patients in time and space. We will map the paths to care for LVOs stroke patients and calculate times based on time of day, day of week, and traffic patterns.

To examine geographic disparities in stroke care there are two methods we will apply. First, we will use spatial scan statistics. These methods generate circles (or ellipses) of various sizes and evaluate observed versus expected rate ratios (risk within circle compared to risk outside) to identify statistically significant “clusters” of outcomes including clustering over time. We will use this approach to find places in the study area where patients are statistically significantly more likely to not get transported directly to an ESC. Spatial scan statics will be estimated using the free software SaTScan. The second method we will use is a structured additive regression model based on a fully Bayesian approach via Markov Chain Monte Carlo (MCMC) simulations. These models can account for spatial and temporal random effects, spatial effects, and spatial-temporal interactions. These models provide geographically smoothed spatial parameter estimates that can be used to visualize disparities in stroke outcomes (e.g., places with a higher odd of not getting transported directly to an ESC) and provide specific location where outcomes are statistically significantly higher or lower (e.g., credible intervals in Bayesian methods) than the study areas average values.

When comparing the outcomes of LVO stroke patients who underwent primary versus secondary outcomes, we will use univariate analysis of the both the primary group and the subgroups. Groups will be compared by X^2^ tests We will then perform multivariate analysis to control for individual stroke centers and initial NIHSS to verify our results. In addition, we will perform a regression analysis to determine the relationship of time to EVT to functional outcomes.

**Fig. 4:**
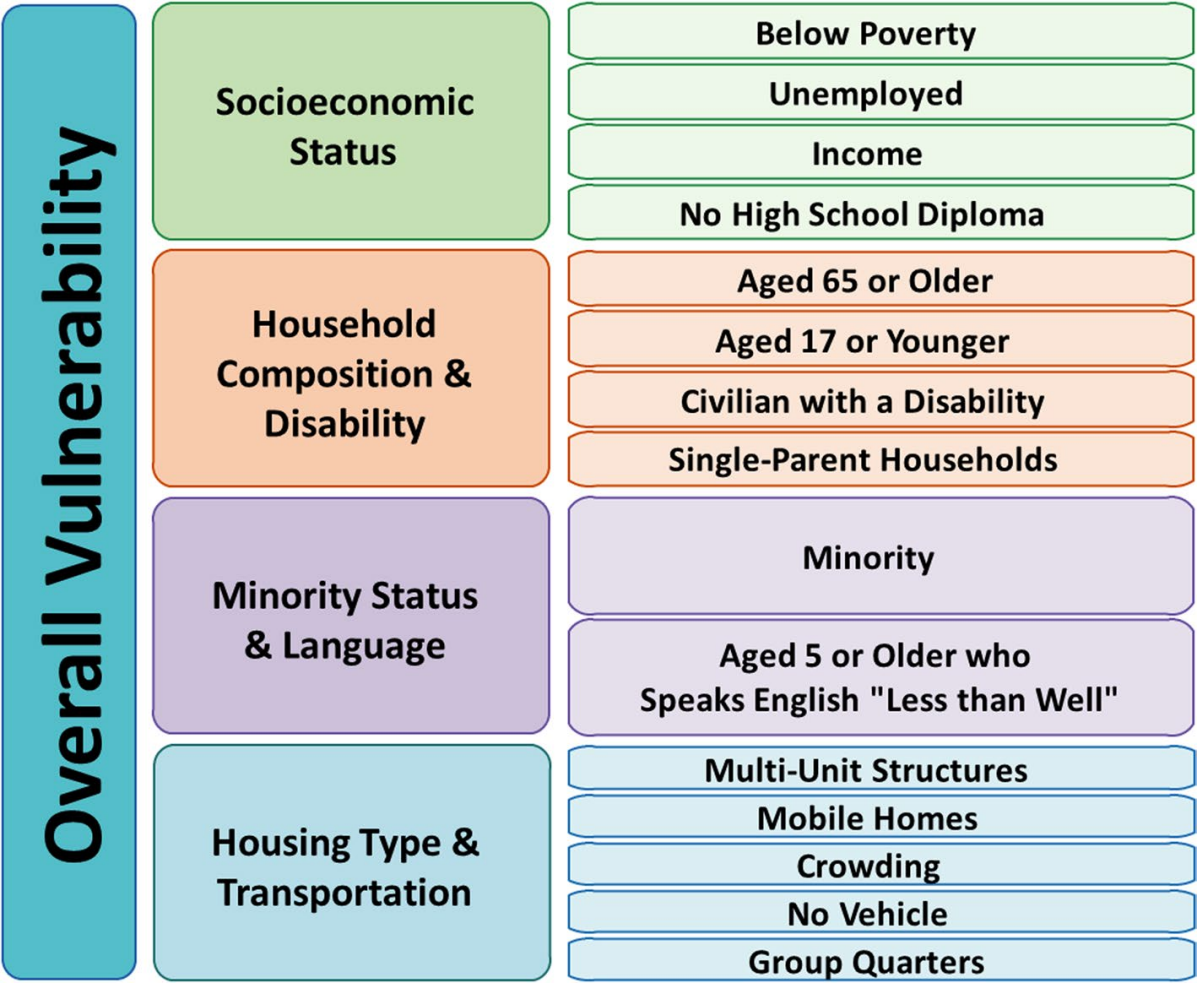
US CDC Social Vulnerability Index

To look at the effect of socioeconomic factors on LVO stroke outcomes, we will use the United States Centers for Disease Control and Prevention (CDC) Social Vulnerability Index (SVI) [23, 24] (Fig. 4). SVI components include socioeconomic status, minority status, and percentage of population over 65 years of age. Because the SVI is available at the level of the census tract, we will plot the SVI components against drive time to ESCs and determine the differences between populations and their access to care.

### Organization, funding, and registration

OPUS-REACH is support by a grant from the American Heart Association. Temple University will serve as the lead site and the other eight health systems will function as spokes of Temple University. The design of the registry as well as the study protocols were developed by the site investigators at all nine health systems. The study was approved by the Institutional Review Boards (IRB) at each health system. Any alternations in the study protocol will be submitted to each individual IRB for review. As this is a registry study, no trial registration is required.

## Discussion

**Fig. 5:**
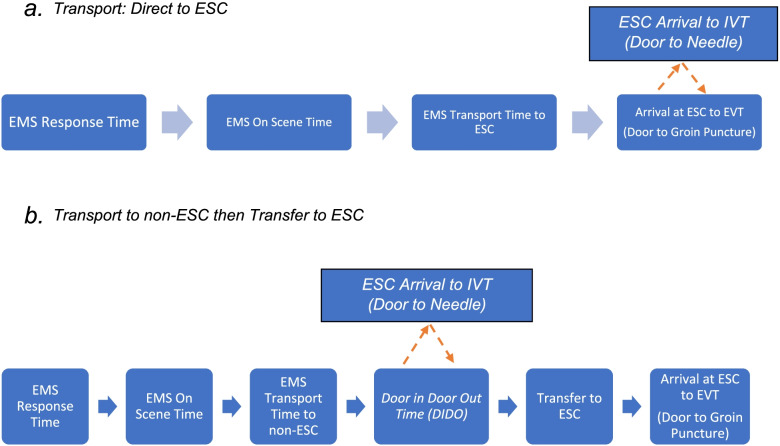
Primary and Secondary Time Intervals

Using the data obtained from the OPUS-REACH registry, we will develop evidenced based algorithms for prehospital transport of LVO stroke patients. We expect that we will find modifiable factors that are associated with poor outcomes in LVO stroke. For example, we hypothesize that outcomes will be worse for patients who were secondarily transferred for EVT rather than directly transported to an ESC. If this is true, then delays may be related to the door-in-door-out time (DIDO) time at the transferring non-ESC (Fig. 5). Long DIDO times be associated with the level of stroke certification for a hospital, whether a hospital is part of an integrated health systems, or the processes for treating and transferring LVO stroke patients. On the other hand, delays in arriving at EVT capable hospitals may not be related to internal stroke processes at referring hospitals but to long transfer times to the ESC. In this case, a prehospital transport algorithm for a suspected LVO stroke patient would include greater emphasis on rapid direct transport including aeromedical transport.

## Conclusion

The OPUS-REACH registry will provide evidence for the creation of a prehospital stroke system of care for LVO patients. Unlike prior published research, we have data spanning from EMS dispatch to ninety day functional outcomes. The OPUS-REACH registry with its breadth of locations, detailed patient records, and multidisciplinary researchers from the fields of emergency medicine, medical geography, and neurology, is poised to design the optimal prehospital systems of care for LVO stroke patients.

## Supplementary Information

**Additional file 1: Supplemental file 1.** Description of the nine health systems in the OPUS-REACH consortium.

## Data Availability

The datasets generated and/or analyzed during the current study are not publicly available due to protected health information but are available from the corresponding author on reasonable request.

## Abbreviations

CDC: United States Centers for Disease Control and Prevention
DIDO: Door-In-Door-Out Time
EMS: Emergency medical services
ESC: Endovascular center
EVT: Endovascular treatment
GIS: Geographical Information Systems
IVT: Intravenous thrombolysis
LVO: Large vessel occlusion
MCMC: Markov Chain Monte Carlo
OPUS-REACH: Optimizing Prehospital Stroke Systems of Care-Reacting to Changing Paradigms
SI: Site Investigator
SVI: Social Vulnerability Index

## References

[1] Virani SS, Alonso A, Benjamin EJ (2020). Heart disease and stroke Statistics-2020 update: a report from the American Heart Association. Circulation.

[2] Centers for Disease Control and Prevention. Underlying Cause of Death CWODA, GA: Centers for Disease Control and Prevention; 2019. Accessed 30 May 2021.

[3] Mokdad AH, Ballestros K, Collaborators USBoD (2018). The state of US health, 1990-2016: burden of diseases, injuries, and risk factors among US states. JAMA.

[4] Berkhemer OA, Fransen PS, Beumer D (2015). A randomized trial of intraarterial treatment for acute ischemic stroke. N Engl J Med.

[5] Jadhav AP, Diener HC, Bonafe A, et al. Correlation between clinical outcomes and baseline CT and CT angiographic findings in the SWIFT PRIME trial. AJNR Am J Neuroradiol. 2017. 10.3174/ajnr.A5406. 10.3174/ajnr.A5406 PMC7963752 29025724

[6] Campbell BC, Mitchell PJ, Kleinig TJ (2015). Endovascular therapy for ischemic stroke with perfusion-imaging selection. N Engl J Med.

[7] Goyal M, Demchuk AM, Menon BK (2015). Randomized assessment of rapid endovascular treatment of ischemic stroke. N Engl J Med.

[8] Jovin TG, Chamorro A, Cobo E (2015). Thrombectomy within 8 hours after symptom onset in ischemic stroke. N Engl J Med.

[9] Albers GW, Marks MP, Kemp S (2018). Thrombectomy for stroke at 6 to 16 hours with selection by perfusion imaging. N Engl J Med.

[10] Kamel H, Parikh NS, Chatterjee A, et al. Access to Mechanical Thrombectomy for Ischemic Stroke in the United States. Stroke. 2021:STROKEAHA120033485. 10.1161/STROKEAHA.120.033485. 10.1161/STROKEAHA.120.033485 PMC8316281 33980045

[11] Jahan R, Saver JL, Schwamm LH (2019). Association Between Time to Treatment With Endovascular Reperfusion Therapy and Outcomes in Patients With Acute Ischemic Stroke Treated in Clinical Practice. JAMA.

[12] Stein LK, Tuhrim S, Fifi J, Mocco J, Dhamoon MS (2019). Interhospital transfers for endovascular therapy for acute ischemic stroke. Stroke.

[13] Shah S, Xian Y, Sheng S (2019). Use, Temporal Trends, and Outcomes of Endovascular Therapy After Interhospital Transfer in the United States. Circulation.

[14] Bogle BM, Asimos AW, Rosamond WD (2017). Regional evaluation of the severity-based stroke triage algorithm for emergency medical services using discrete event simulation. Stroke..

[15] Froehler MT, Saver JL, Zaidat OO, et al. Interhospital transfer prior to Thrombectomy is associated with delayed treatment and worse outcome in the STRATIS registry. Circulation. 2017. 10.1161/circulationaha.117.028920. 10.1161/CIRCULATIONAHA.117.028920 PMC5732640 28943516

[16] Holodinsky JK, Williamson TS, Demchuk AM (2018). Modeling stroke patient transport for all patients with suspected large-vessel occlusion. JAMA Neurol.

[17] Venema E, Burke JF, Roozenbeek B (2020). Prehospital Triage Strategies for the Transportation of Suspected Stroke Patients in the United States. Stroke.

[18] Harris PA, Taylor R, Thielke R, Payne J, Gonzalez N, Conde JG (2009). Research electronic data capture (REDCap)--a metadata-driven methodology and workflow process for providing translational research informatics support. J Biomed Inform.

[19] Rankin J (1957). Cerebral vascular accidents in patients over the age of 60. III. Diagnosis and treatment. Scott Med J.

[20] Banks JL, Marotta CA (2007). Outcomes validity and reliability of the modified Rankin scale: implications for stroke clinical trials: a literature review and synthesis. Stroke.

[21] van Swieten JC, Koudstaal PJ, Visser MC, Schouten HJ, van Gijn J (1988). Interobserver agreement for the assessment of handicap in stroke patients. Stroke..

[22] Prus N, Isenberg D, Ramsey F, Almeda-Lopez A, Arandela K, Watts, S. Gentile, N, Hospital Retrospective Determination of the Modified Rankin Scale Score from Patient Charts. Oral ePoster Presentation. Denver: Society for Academic Emergency Medicine; 2020.

[23] Flanagan BE, Hallisey EJ, Adams E, Lavery A (2018). Measuring community vulnerability to natural and anthropogenic hazards: the Centers for Disease Control and Prevention's social vulnerability index. J Environ Health.

[24] Flanagan BE, Gregory EW, Hallisey EJ, Heitgerd JL, Lewis BP. A social vulnerability index for disaster management. J Homeland Secur Emerg Manage. 2011;8(1). 10.2202/1547-7355.1792.

