# Correction: Optimizing Prehospital Stroke Systems of Care-Reacting to Changing Paradigms (OPUS-REACH): a pragmatic registry of large vessel occlusion stroke patients to create evidence-based stroke systems of care and eliminate disparities in access to stroke care

**DOI:** 10.1186/s12883-022-02695-1

**Published:** 2022-05-07

**Authors:** Derek L. Isenberg, Kevin A. Henry, Adam Sigal, Traci Deaner, Jason T. Nomura, Kathleen A. Murphy, Derek Cooney, Susan Wojcik, Ethan S. Brandler, Alexander Kuc, Gerard Carroll, Chadd K. Kraus, Judy B. Shahan, Joseph Herres, Daniel Ackerman, Nina T. Gentile

**Affiliations:** 1 Department of Emergency Medicine, Lewis Katz School of Medicine at Temple University, Philadelphia, PA USA; 2 Department Geography and Urban Studies, Temple University, Philadelphia, PA USA; 3 Department of Emergency Medicine, Tower Health, Reading, PA USA; 4 Department of Emergency Medicine, ChristianaCare, Newark, DE USA; 5 Department of Emergency Medicine, State University of New York-Upstate Campus, Syracuse, NY USA; 6 Deparement of Emergency Medicine, Stony Brook Medicine, Stony Brook, NY USA; 7 Department of Emergency Medicine, Cooper Medical School of Rowan University, Camden, NJ USA; 8 Department of Emergency Medicine, Geisinger, Danville, PA USA; 9 Department of Emergency Medicine, Einstein Healthcare Network, Philadelphia, PA USA; 10 Department of Neurology, St. Luke’s University Health Network, Bethlehem, PA USA

**Correction to: BMC Neurol 22, 132 (2022)**

**https://doi.org/10.1186/s12883-022-02653-x**

Following publication of the original article [1], the authors identified an error in the author name of Chadd K. Kraus.

The incorrect author name is: Chadd Krauss

The correct author name is: Chadd K. Kraus

The author group has been updated above and the original article [1] has been corrected.

The original article [1] has been updated.
