# Supplementary material for: Optimizing Prehospital Stroke Systems of Care-Reacting to Changing Paradigms (OPUS-REACH): a pragmatic registry of large vessel occlusion stroke patients to create evidence-based stroke systems of care and eliminate disparities in access to stroke care
Source: BMC Neurol. 2022 Apr 7;22:132. doi: 10.1186/s12883-022-02653-x (PMC8988419; doi:10.1186/s12883-022-02653-x)
Supplement: Supplementary file 1 — Additional file 1: Supplemental file 1. Description of the nine health systems in the OPUS-REACH consortium. [file 12883_2022_2653_MOESM1_ESM.docx]

1. **Temple University Health System** consists of three hospitals located in Philadelphia, Pennsylvania. The three hospitals see approximately 200,000 patients per year throughout the three emergency departments. Temple University Hospital is a certified comprehensive stroke center while Episcopal Hospital and Jeanes Hospital are both certified as primary stroke centers. Throughout the system, TUHS cares for approximately 500 stroke patients per year and performs approximately 40 endovascular procedures for acute ischemic stroke annually.
2. **Einstein Healthcare Network** is comprised of three acute care hospitals, Einstein Medical Center – Philadelphia, Einstein Medical Center – Elkins Park, Einstein Medical Center – Montgomery, all of which are designated primary stroke centers. Einstein Medical Center Philadelphia is a 548-bed tertiary-care center and is the largest independent academic medical center in Philadelphia. Einstein Healthcare Network serves over 620,000 outpatient visits a year, over 42,000 hospital admissions, and over 162,000 emergency room visits/year. Einstein Medical Center Philadelphia is also recognized by the Joint Commission as a thrombectomy-Capable Stroke Center (TSC) in collaboration with the American Heart Association/American Stroke Association (AHA/ASA). There are over 650 stroke activations annually within Einstein Healthcare Network and Einstein Medical Center Philadelphia performs over 40 mechanical thrombectomies annually for acute stroke cases.
3. **Cooper University Hospital (CUH)**  is an academic medical center located in Camden, New Jersey. It is the only Level 1 trauma center in southern New Jersey. The Emergency Department at CUH treats over 80,000 patients annually. The Department of Emergency Medicine has a dedicated research director along with multiple study coordinators. Cooper University Health Care has earned The Joint Commission’s Gold Seal of Approval and the American Heart Association/American Stroke Association’s Heart-Check mark for Advanced Certification for Comprehensive Stroke Centers. Cooper University Hospital provides Emergency Medical Services (EMS) for the City of Camden with an annual volume of approximately 40,000 calls. The ground fleets consist of 11 EMS vehicles and four physician response vehicles. The Department of Emergency Medicine also sponsors an EMS fellowship.
4. **Tower Health System**, headquartered in Reading, Pennsylvania provides care to 2.5 million patients. The flagship facility, Reading Hospital, is a 647 acute care facility located in Berks County, Pennsylvania. The Reading Hospital Emergency Department, with an annual volume of over 136,000 patients, is the busiest Emergency Department in Pennsylvania. The Emergency Department has a strong relationship with the EMS agencies in the area. Many of the pre-hospital personnel in Berks County trained at the Reading Hospital School of Health Science under the mentorship of the emergency medicine faculty.  Over 90% of the EMS agencies have Reading Hospital Emergency Medicine attendings serving as Medical Directors.  Reading Hospital is a thrombectomy capable hospital and received the American Heart Association/American Stroke Association 2021 Get with The Guidelines–Stroke Gold Plus Quality Achievement Award (Target: Stroke Advanced Therapy).   The Reading Hospital cares for approximately 725 acute strokes a year with about 78% ischemic.  In 2020, the hospital administered tPA to 65 patients and performed EVT on 55 patients.  In addition, Reading Hospital performed coiling or clipping on 14 patients with acute subarachnoid hemorrhages. 5 network hospitals refer patients to the Reading Hospital.
5. **Geisinger**: Geisinger, headquartered in Danville, Pennsylvania, is an integrated health services organization recognized for its role in the development of innovative care delivery models. Geisinger serves more than 3 million residents in a catchment area including 45 counties in Pennsylvania, including a largely rural and underserved population, with 29 counties designated as rural. Geisinger has 12 hospital campuses, two research centers and a 580,000-member health plan. Geisinger Medical Center (GMC) in Danville, Pennsylvania is the flagship hospital for the system, with a second tertiary site, Geisinger Wyoming Valley (GWV) located in Wilkes Barre, PA. GMC is central Pennsylvania’s only Level I Trauma Center and its only compressive stroke center (CSC). GWV is a Level II Trauma Center and CSC. Geisinger Health System Cares for approximately 2000 stroke patients per year and performs 160 mechanical thrombectomies per year. Geisinger LifeFlight provides air and critical care ground transportation for stroke patients between primary stroke centers and GMC and GWV. The EMS Medical Director for the Commonwealth of Pennsylvania is faculty at GMC.
6. **State University of New York-Upstate (SUNY-Stony Brook)**is a 624 bed hospital located in Stony Brook, New York. SBUH cares for approximately 103,000 patients a year in its ED. In May 2018, SBUH Hospital became the first hospital in Suffolk County, New York to achieve Comprehensive Stroke Center certification. The Emergency Medicine Research Center (EMRC) is located within the Department of Emergency Medicine at Stony Brook University and Medical Center. The Emergency Medical Services Division at SBUH includes 80 paramedics and EMTs who provide critical care transport service from community hospitals to SBUH. Approximately 9,000 patients are transported each year. In April 2019, Stony Brook Medicine launched Long Island’s first Mobile Stroke Unit Program.
7. **St. Luke’s University Health Network (SLUHN)** is a non-profit, regional, fully integrated, health care network providing services at twelve hospitals and more than 300 sites in Eastern Pennsylvania and New Jersey. Across the health system the emergency departments treat more than 390,000 patients per year and as a network in the last year 1334 patients with stroke were cared for at their hospitals . The flagship facility is the 480 bed St. Luke’s University Campus in Bethlehem, Pennsylvania which is designated as a comprehensive stroke center and performed 83 thrombectomies for stroke related to large vessel occlusion in the last year. St. Luke’s University Health Network supports a multidisciplinary emergency response team for the management of acute stroke patients as well as a team of health care providers dedicated to the ongoing care of stroke victims.
8. **ChristianaCare** is a non-profit, regional, fully integrated, health care network providing services in Northern Delaware. The network consists of two hospitals and one free standing ED. Christiana Hospital, the flagship hospital of the system, is the only adult level I trauma center, only comprehensive stroke center and has the only dedicated neurocritical care unit in Delaware. Along with Delaware, Christiana Hospital provides tertiary care to parts of Pennsylvania, New Jersey and Maryland. Across the system, ChristianaCare averages nearly 200,000 annual Emergency Department visits.  The neurosciences program at ChristianaCare has over 1500 stroke hospitalizations and performs over 150 acute endovascular stroke cases annually.  With our partners across the region we receive several hundred transfers to provide advanced neuroscience care.  The system also consistently achieves high quality measure with American Stroke Association Gold and Target Stroke Awards.

1. The **State University of New York- Upstate Campus (SUNY-Upstate)** is an academic medical center located in Syracuse, New York. Hospital system consists of a community hospital along with a regional level 1 trauma center. Across the health system, SUNY-UPSTATE cared for 111,233 emergency department patients in 2019. SUNY-Upstate is a certified comprehensive stroke center providing tele-stroke services to many smaller hospitals in the area. SUNY-Update cares for approximately 1900 stroke patients per year and performs 87 mechanical thrombectomy’s annually. SUNY-Upstate hosts an emergency medicine residency as well as a fellowship in emergency medical services. The department provides EMS physician field response as well as medical direction for the largest advanced life support service in the area.
